# Effects of corn supplementation on serum and muscle microRNA profiles in horses

**DOI:** 10.1002/fsn3.3259

**Published:** 2023-02-14

**Authors:** Clarissa Carver, Jason Bruemmer, Stephen Coleman, Gabriele Landolt, Tanja Hess

**Affiliations:** ^1^ Animal Sciences Department Colorado State University Fort Collins Colorado USA; ^2^ USDA APHIS WS NWRC Fort Collins Colorado USA; ^3^ Clinical Sciences Department Colorado State University Fort Collins Colorado USA

**Keywords:** corn, horse, microRNA, muscle, serum

## Abstract

Laminitis associated with equine metabolic syndrome causes significant economic losses in the equine industry. Diets high in non‐structural carbohydrates (NSC) have been linked to insulin resistance and laminitis in horses. Nutrigenomic studies analyzing the interaction of diets high in NSCs and gene expression regulating endogenous microRNAs (miRNA) are rare. This study's objectives were to determine whether miRNAs from dietary corn can be detected in equine serum and muscle and its impacts on endogenous miRNA. Twelve mares were blocked by age, body condition score, and weight and assigned to a control (mixed legume grass hay diet) and a mixed legume hay diet supplemented with corn. Muscle biopsies and serum were collected on Days 0 and 28. Transcript abundances were analyzed using qRT‐PCR for three plant‐specific and 277 endogenous equine miRNAs. Plant miRNAs were found in serum and skeletal muscle samples with a treatment effect (*p* < .05) with corn‐specific miRNA being higher than control in serum after feeding. Endogenous miRNAs showed 12 different (*p* < .05) miRNAs in equine serum after corn supplementation, six (eca‐mir16, ‐4863p, ‐4865p, ‐126‐3p, ‐296, and ‐192) previously linked to obesity or metabolic disease. The results of our study indicate that dietary plant miRNAs can appear in circulation and tissues and may regulate endogenous genes.

## Graphical abstract

Supplementing corn to horses has resulted in increases in corn microRNA in horses' serum. Corn supplementation induced changes in endogenous micro RNAs related to insulin and glucose metabolism.

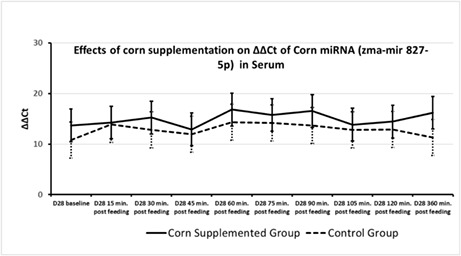

## 1 INTRODUCTION

The diet and feeding behaviors of horses today are dramatically different from those of their ancestors and tailored to human convenience, not physiologic make‐up. Many equine rations contain energy supplements like corn, which is a high‐energy feed and low‐cost ingredient. Corn and other feeds containing non‐structural carbohydrates (NSC) have been linked to a variety of equine metabolic conditions including laminitis, insulin resistance, and obesity (Pollitt & Visser, 2010). Together, these conditions may indicate the horse has equine metabolic syndrome (EMS), a condition with similarities to metabolic syndrome in humans (Ertelt et al., 2014; Selim et al., 2015). These issues and conditions are not yet fully understood, and investigations into how dietary factors can influence the regulation of gene expression are being conducted to identify reliable markers for the diagnosis of these conditions. The role of microRNAs (miRNAs) in gene regulation and as potential disease markers has been subject to recent and current research (da Costa Santos et al., 2018; Nulton, 2014), including the influence of plant‐derived miRNAs acquired from the diet (Zhang et al., 2012).

MicroRNAs are small non‐coding molecules approximately 18–22 nucleotides in length that affect post‐transcriptional regulation of gene expression. MicroRNAs can be found circulating freely in blood or associated with micro‐vesicles (Zhang et al., 2012). Regulation of gene expression occurs through inactivation when the miRNAs bind to the target mRNA (Zaiou et al., 2018). Nearly all cell functions are thought to involve miRNAs in their regulation. The miRNAs circulating in the blood indicate the influence these molecules can have on the regulation of cells, tissues, and physiological processes throughout the entire body (Mittelbrunn & Sánchez‐Madrid, 2012). MicroRNAs exert their function by gene regulation through gene silencing, translational activation, transcriptional, and post‐transcriptional gene regulation (O'Brien et al., 2018). MicroRNAs are present in biological fluids, and their release in extracellular compartments is thought to be a regulated process. Some miRNAs regulate the activity of smooth muscles, in cancer they can promote metastasis. MicroRNAs can serve as biomarkers of disease and play an important role in cell communication. Extracellular miRNAS are active in recipient cells and interact with cell surface receptors and are considered to have hormone‐like activities (O'Brien et al., 2018). Interest in miRNAs and their roles within diseases and cellular processes has led to increased research in both healthy (Zhang et al., 2012) and diseased individuals of many species such as mice, rats, horses, and humans (Bhaskaran & Mohan, 2014). Other studies have suggested a novel idea that diet‐derived exogenous plant miRNAs are transferred to blood and can be transported to tissues of vertebrates (Zhang et al., 2012; Zhou et al., 2009). There is a potential of food‐derived miRNAs to influence mammal endogenous genes.

The objective of this study was to evaluate the uptake of diet‐derived plant and corn miRNAs in horses. More specifically, the possible influence ingestion of corn and forage can have on equine body miRNAs was also investigated. In this study, we compare the effects of a corn‐supplemented diet compared with a control diet (grass and alfalfa only).

## 2 MATERIALS AND METHODS

### 2.1 Animal care and feeding protocols

**FIGURE 1:**
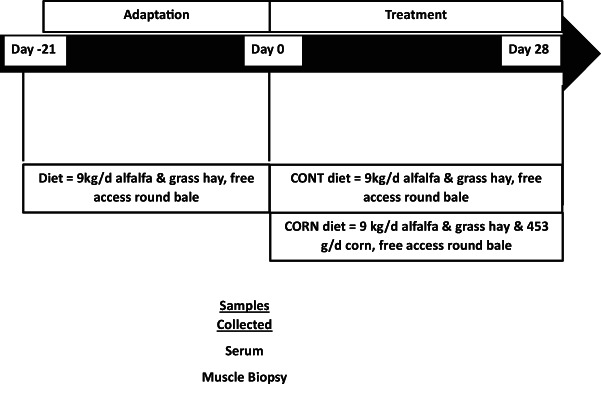
Schematic sample collection during the study. Horses were placed in the same pen for a 21‐day adaptation period before the 28‐day feeding trial. Serum and muscle biopsies were collected on Days 0 and 28.

All experiments were approved by the Colorado State University Institutional Animal Care and Use Committee (Protocol #18‐7961A). Twelve Quarter‐horse type mares (quarter horses or its crosses) aged 10–18 years were used for this study. All horses were maintained together on a dry lot with free‐choice water and hay for a 21‐day adjustment period before the 28‐day trial period. Weight and body condition scores (BCS), using the Henneke scale of 1–9, were recorded before the start of treatment. Body condition scores assess the relative fat content in horses. Horses were blocked by age, BCS, and body weight and assigned to either the control group (CONTROL; 15.8 ± 0.7 years, 553.9 ± 13.0 kg, BCS 6.0 ± 0.2) or the corn‐supplemented diet group (CORN; 14.3 ± 0.7 years, 555.1 ± 13.0 kg, BCS 6.2 ± 0.2) with an *n* = 6 per treatment group. For the trial period horses were fed once daily in the morning. The diets were formulated to meet or exceed the minimum nutritional requirements for maintenance (National Research Council, 2007) and were analyzed to determine nutritional composition (Table 1). Horses were fed hay from the feed bunk once a day and had free access to grass hay round bales for 21 days before the study started. On Day 0, all horses had a basal blood sample and muscle biopsies taken (Figure 1). Horses had access to free choice hay the night prior to the Day 0 morning collections. Starting on Day 0, after blood sample collection, horses on the corn‐supplemented diet were gradually acclimated to the diet over 8 days with individual feed bags being utilized to ensure each horse had the correct amount of corn offered to them at each feeding.

**TABLE 1:** Feed analyses on dry matter basis^a^.

Analysis parameters	Round bale	Chopped hay mixture	Steam flaked corn
Dry matter, %	93.4	90.6	86.4
Digestible energy, Mcal/kg	2.21	2.18	3.96
Crude protein, %	7.2	17.2	7.5
Estimated lysine, %	0.25	0.60	0.21
Lignin, %	3.1	5.0	–
Acid detergent fiber, %	35.2	34.5	2.4
Neutral detergent fiber, %	58.3	52.5	6.2
Water soluble carbohydrates, %	14.0	8.7	2.1
Ethanol soluble carbohydrates, %	5.9	7.1	1.5
Starch, %	0.9	0.4	78.6
Non‐fiber carbohydrates, %	24.5	17.7	80.7
Crude fat, %	2.6	3.0	–
Ash, %	7.4	9.7	–

^a^ Analyzed by Equi Analytical.

On Days 1–3 horses were fed 113 g of corn at each feeding, on Days 4 and 5 the horses were fed 226 g per feeding, and Days 6–7 340 g per feeding. Horses received the full 453 g of steam flaked corn from Day 8 onward. During the 28 days of supplementation, the control group was offered 9 kg/horse/day of chopped mixed alfalfa and grass hay daily in a feed bunk with enough area for each horse, while the corn‐supplemented group was offered 9 kg/horse/day of chopped mixed alfalfa and grass plus 453 g/horse/day of steam‐flaked corn fed in individual nose bags. Both groups also had access to free choice grass hay from a round bale and a salt block plus ad libitum water. The difference in energy (about 1 Mcal) was made up by CONTROL horses consuming more hay. The blood collection for the post‐ingestion of corn started, immediately after the supply of 453 g of corn for the CORN and fasting in the CONTROL group on Day 28. Both groups were placed in individual stalls during blood collection and had access to water. Horses had access to hay during the night before the blood and muscle biopsies.

### 2.2 Blood collection and serum processing

Two 10 ml blood samples were collected in serum separator blood tubes (Becton, Dickinson and Company, product #367985) at each time point. Blood was collected on Days 0 and 28 of the feeding trial period. On Day 0 samples were collected by jugular venipuncture via a vacutainer tube once in the morning. Horses had free access to hay before blood collection. The Day 28 samples were collected from jugular intravenous catheters at 0, 15, 30, 45, 60, 75, 90‐, 105‐, 120‐, and 360‐min post‐feeding. In a prior study (Nulton, 2014) blood samples were only taken 360 min after the feeding of corn, rice, or alfalfa hay. Figure 1 illustrates sample collection across the duration of the study. The frequency and timing of post‐feeding samples were chosen to trace the appearance of miRNA from steam flaked corn (Nulton, 2014) in blood. The intravenous catheters were placed using aseptic technique and secured with a suture. After each blood sample collection, the lines and catheters were flushed with heparinized saline to avoid clotting and clamped. Blood samples were then allowed to clot at room temperature for 30 min at a 45° angle before centrifugation for 10 min at 2000 *g* at 4°C to separate serum. The serum was removed, transferred to polypropylene tubes, and stored at −80°C until RNA isolation. Blood collected at each time point was analyzed to determine whether plant‐specific miRNAs were detectable in serum. Endogenous equine miRNAs were analyzed in samples from Day 0, and on Day 28 samples that were taken 60 and 360 min after feeding (Figure 1). The 60‐min time was selected as it was the midpoint of the 2‐h sampling window, and the 360 min was chosen to allow comparison to a previous equine study (Nulton, 2014).

### 2.3 Muscle biopsy collection

Muscle biopsies were taken from the *Gluteus medius* muscle of each horse before (Day 0) immediately before blood samples were collected, and similarly at the completion (Day 28) of the trial period in the morning prior to feeding corn to the treatment group. Biopsies were taken from the left side of all the horses on Day 0 and the right side on Day 28, opposite sides were chosen to minimize tissue trauma and avoid any scar tissue from the previous biopsy. Figure 1 illustrates sample collection across the duration of the study. Horses were placed in stocks and the biopsy site was prepared by shaving the hair and scrubbing the site with betadine and alcohol, before local anesthesia of the area through subcutaneous administration of 1 ml of 2% lidocaine (VetOne). Tissue biopsies were collected aseptically, after incision with a scalpel, by inserting a Bergstrom biopsy needle (Popper and Sons, 6 mm external diameter, 9 cm length) midway between the tuber coxae and ischiatic tuberosity to a depth of 6 cm. Samples were placed in sterile cryotubes (2 ml, Life Science Products), snap‐frozen in liquid nitrogen, and stored at −80°C until RNA isolation.

### 2.4 RNA isolation from serum

RNA was isolated from the collected serum samples using a modified version of the manufacturer's suggested protocol for TRI Reagent BD (Molecular Research Center, Inc.) in duplicate and combined after RNA isolation to concentrate the sample. Briefly, 500 μl of serum was combined with 10 μl polyacryl carrier and 750 μl of TRI Reagent BD for sample lysis. After lysis, 200 μl of chloroform was added, vortexed to mix, and allowed to sit at room temperature for 15 min before centrifuging for 10 min at 12,000 *g* at 4°C. The aqueous upper phase, which contained the RNA, was transferred to a clean 1.5 ml tube and precipitated with 500 μl isopropanol and 100 μl sodium acetate (pH 5.4) overnight at −20°C. The sodium acetate was added to the protocol for serum samples to precipitate out more RNA as lower amounts than desired were yielded when not included in the protocol. Precipitated RNA was pelleted by centrifugation for 10 min at 12,000 *g* at 4°C. The supernatant was discarded, and the pellet washed three times with 1 ml of 75% ethanol. The sample was air dried and rehydrated with 10 μl RNase‐free water then combined with the duplicate sample for a total of 20 μl. The concentration and purity were determined with a Nanodrop Spectrophotometer ND‐2000 (Thermo Scientific).

### 2.5 RNA isolation from muscle tissue

RNA isolation from skeletal muscle was accomplished by first grinding the samples with a mortar and pestle that had been previously washed with ethanol and nanopure water to reduce chances of contamination. The mortar and pestle were then conditioned with liquid nitrogen and the entire muscle sample was placed into liquid nitrogen within the mortar and ground into a fine powder. Approximately 2 g of powder was then transferred to a clean 1.5 ml tube and 750 μl TRIzol (Thermo Scientific) was added according to the manufacturer's protocols and vortexed to lyse the cells within the sample. After the cell lysing, 200 μl of chloroform was added to the sample, mixed by vortexing, and incubated at room temperature for 15 min followed by centrifugation for 10 min at 12,000 *g* at 4°C. The aqueous supernatant containing the RNA was then transferred to a clean 1.5 ml tube, combined with 500 μl of isopropanol, and stored overnight at −20°C, followed by centrifugation for 10 min at 12,000 *g* to collect the precipitated RNA. The pellet was washed with 1 ml of 75% ethanol, air dried, and resuspended in 20 μl of nuclease‐free water. The samples were then tested on a Nanodrop Spectrophotometer ND‐2000 (Thermo Scientific) to determine RNA purity and concentration. All samples isolated from the muscle biopsies were treated with DNA‐ free DNase Treatment and Removal Reagent (Thermo Scientific, Product #AM1906) to remove genomic DNA, and RNA purity and concentration were re‐examined before proceeding. Samples were stored at −80°C until they were used for expression analysis.

### 2.6 Plant RNA isolation

RNA was isolated from 2 g of the hay and corn samples using TRI Reagent (Molecular Research Center, Inc.) following a modified version of the manufacturer's protocol. The sample was pulverized using a mortar and pestle (see above cleaning protocol), combined with 750 μl of TRI reagent in a 1.5 ml tube, and homogenized by vortexing. After this, 200 μl of chloroform was added, then the samples were vortexed and centrifuged at 12,000 *g* for 10 min to separate the phases, and extracellular debris was removed. The supernatant was transferred to a new tube and the RNA was precipitated by the addition of 250 μl isopropanol and 25 μl sodium acetate solution (pH 5.4) and stored overnight at −20°C. The samples then were centrifuged for 10 min at 12,000 *g* and the pellet washed three times with 75% ethanol. The samples were treated with a DNA‐free DNase treatment to remove any genomic DNA (Thermo Scientific, Product #AM1906) and RNA purity assessed with a Nanodrop Spectrophotometer ND‐2000 (Thermo Scientific) (da Costa Santos et al., 2018). Samples were then stored at −80°C until cDNA synthesis.

### 2.7 Reverse transcription

cDNA was generated for each RNA sample using the miScriptII RT kit (Qiagen). Per the manufacturer's instructions, 4 μl 5× HiSpec Buffer, 2 μl 10× nucleics mix, 1000 ng total RNA, 2 μl of reverse transcriptase mix, and nuclease‐free water, were combined for a total volume of 20 μl for each reaction and placed in a Bio‐Rad T100 Thermal Cycler (Bio‐Rad Laboratories). The reaction was incubated for 60 min at 37°C, then 95°C for 5 min. Complete reactions were diluted with 100 μl of nanopure water for a total volume of 120 μl and a cDNA concentration of 500 pg/μl and stored at −20°C.

### 2.8 Quantitative reverse‐transcriptase PCR analysis

Transcript expression was determined by quantitative reverse‐transcriptase PCR (qRT‐PCR) using the QuantiTect SYBR Green PCR kit (Qiagen) for a total of 277 endogenous mature equine miRNA transcripts. This panel was previously used for other equine studies (da Costa Santos et al., 2018; Nulton, 2014; Santos et al., 2021) and was based off in silico prediction of equine miRNAs sequences (Zhou et al., 2009). Three mature plant miRNA transcripts, ath‐miR156a, zma‐miR827‐5p, and osa‐miR1866‐3p, were also included. Ath‐miR156a is conserved across alfalfa and corn, zma‐miR827‐5p is unique to corn, and osa‐miR1866‐3p is unique to rice. These three plant miRNA transcripts were selected for analysis based on previous reports of their expression in serum, corn, rice, or plants in general (Jiao et al., 2011; Xue et al., 2009; Zhang et al., 2009, 2012). The primers for these specifically targeted the sequences of RNA for these analyzed miRNAs. These three plant miRNA transcripts were also used in a previous equine study with ath‐miR156a being the only one detected in equine serum and tissue post‐feeding (Nulton, 2014). The relative level of the mature plant miRNA transcripts was assessed in total equine serum and skeletal muscle at all time points. The primer sequences were ascertained from published plant genomes. Furthermore, the primer sequence for the plant‐specific miRNA were BLASTed against equine genome with no matches detected. The primer sequences were ascertained from published plant genomes. Furthermore, the primer sequence for the plant specific miRNA were BLASTed against equine genome with no matches detected.

The relative abundance of the endogenous equine transcripts was determined for all muscle samples, Day 0 serum, Day 28 serum 60, and 360 min after feeding. For plant transcripts, each real‐time PCR reaction contained 6 μl of total reaction, including 3 μl 2× QuantiTect SYBR Green PCR Master Mix, 0.60 μl 10× miScript Universal reverse primer, 1.15 μl of nuclease‐free water, 0.25 μl of cDNA, and 1 μl miRNA specific forward primer. For the equine endogenous transcripts, all elements for each 6 μl reaction were the same, except 1.28 μl of nuclease‐free water and 0.12 μl of cDNA were used. Cycle conditions consisted of reaction initiation at 95°C for 15 min, followed by 45 cycles of 94°C for 15 s to denature, 55°C for 30 s to anneal, and 70°C for 30 s for extension. Plates were run in duplicate, and miRNA transcripts were considered present if they were present at a cycle number <37 and confirmed with amplification curves and singular melt peaks (da Costa Santos et al., 2018). Negative RT control, non‐template control, and positive RNU1 control were included on all plates.

### 2.9 Statistical analysis

To determine whether plant‐ and corn‐specific miRNA transcripts were present within equine serum and muscle, raw Ct values were first normalized to RNU1, a small RNA used as a reference gene in expression studies of human serum samples (Sanders et al., 2012). The Ct values of RNU1 had a standard deviation of <2.5 cycles across all samples. The normalized Ct values for the plant, corn, and rice transcripts were considered ΔCt (ΔCt = raw Ct−RNU1 Ct). Then, ΔΔCt in serum was calculated as the difference between ΔCt of the different timelines and the basal sample, meaning that each horse was its own control at Day 0. Muscle ΔΔCt was calculated as the difference in ΔCt at Day 28 and ΔCt at Day 0.

The raw Ct values for the endogenous equine miRNA transcripts in the serum samples were normalized to the geometric mean of 3 endogenous controls (eca‐mir376c, eca‐mir14030, and eca‐mir770) that all had individual standard deviations of <1.1 cycles (Mestdagh et al., 2009). The raw Ct values from the muscle samples were also normalized to the geometric mean of 3 endogenous controls (eca‐mir15a, eca‐mir6155p, and eca‐mir770), which had standard deviations of <2 cycles amongst all muscle samples (Mestdagh et al., 2009). Only miRNAs detected in all samples at all time points were used for analysis. Normalized Ct values were represented as ΔCt (ΔCt = raw Ct−geometric mean Ct), and the ΔCt for each gene on Day 0 was then used as the calibrator for calculating ΔΔCt (ΔΔCt = ΔCt−calibrator) allowing each horse to act as its' control. ΔΔCt was used to determine changes in transcript abundance.

Normal Q‐Q plots were used to check visually for normal distribution in R (version 3.5 statistical software). Dependent variables (body weight, BCS, ΔΔCt) were analyzed using a mixed model within the SAS statistical software (9.4 version), with significance set at a *p*‐value ≤ .05. Independent variables for serum analysis were treatment, time, and its interactions; horse by treatment was a random effect included in the analysis model. Significant results compared with least square means analysis and results presented as means ± SE. For the serum corn, rice, and plant miRNA times were 0, 15, 30, 45, 60, 75, 90, 105, 120, and 360 min. For the endogenous miRNA, time was 60 and 360 min, Figure 1. For the muscle miRNA analysis, a simple ANOVA was performed with time and treatment and interactions as independent variables, time being Days 0 and 28 of supplementation.

## 3 RESULTS

### 3.1 Body weight, body condition scores, and feed miRNA content

Horses were weighed and body condition scored on the Henneke scale from 1 to 9 on Days 0 and 28. No time, treatment, or time by treatment effect was found (Table 2).

**TABLE 2:** Horse body condition score and weight.

Group	Body condition score^a^	Bodyweight, kg
CORN Day 0	6.17 ± 0.41	553.91 ± 26.90
CONTROL Day 0	6.00 ± 0.58	552.78 ± 35.84
CORN Day 28	6.17 ± 0.61	582.11 ± 43.50
CONTROL Day 28	6.08 ± 0.74	567.07 ± 33.07

*Note*: Horses were weighed on a livestock scale on Days 0 and 28 of the trial. Shown as group mean and SD, *n* = 6 horses/trt.

^a^ Henneke body condition score system, score 1–9.

Analysis of the dietary corn and hay detected ath‐miR156a transcripts in all feed components. Osa‐miR1866‐3p and zma‐miR827‐5p were only detectable in the corn feed (Table 3). The highest concentration of the corn‐specific miRNA (Zma‐miR827‐5p) was found in steam flaked corn (Table 3). Osa‐mir1866‐3P, the rice‐specific miRNA, was found in steam flaked corn at very low concentration (Table 3) shown by the raw CT.

**TABLE 3:** Plant miRNA expression across feeds.

miRNA	RNA sample	Ct
Ath‐miR156a	Mixed grass hay round bale	33.3
Ath‐miR156a	Chopped mixed grass hay	34
Ath‐miR156a	Steam flaked corn	26.3
Zma‐miR827‐5p	Mixed grass hay round bale	Undetectable
Zma‐miR827‐5p	Chopped mixed grass hay	Undetectable
Zma‐miR827‐5p	Steam flaked corn	23.4
Osa‐mir1866‐3P	Mixed grass hay round bale	Undetectable
Osa‐mir1866‐3P	Chopped mixed grass hay	Undetectable
Osa‐mir1866‐3P	Steam flaked corn	36

*Note*: Levels of plant miRNAs were detected using qRT‐PCR in the round bales, chopped hay, and corn fed to horses throughout the feed trial. Ct indicates the cycle threshold.

### 3.2 Diet‐derived plant miRNAs

**FIGURE 2:**
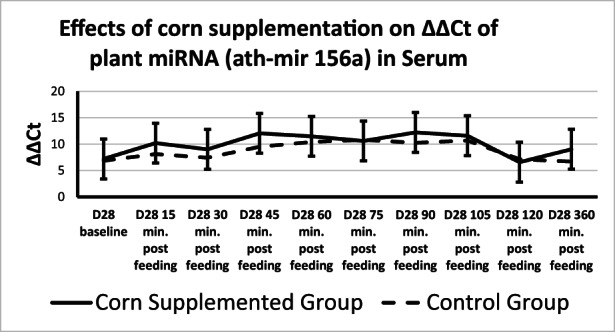
Level of Plant miRNA (ath‐miR156a) in serum across time. qRT‐PCR was performed to determine levels of ath‐miR156a in serum across all diets at Days 0 and 28. No treatment or time by treatment effect was found. *p*‐value ≤ .05. Shown as group means and SD. Data were normalized to RNU1.

**FIGURE 3:**
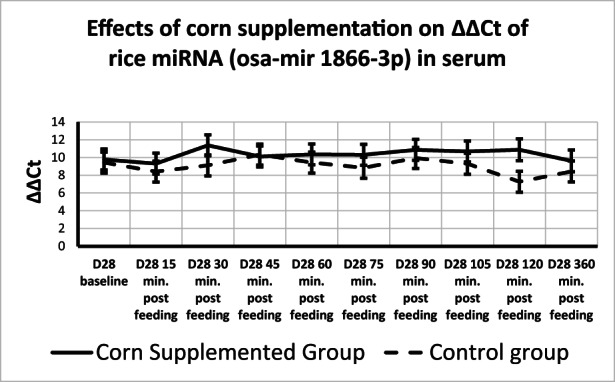
Level of osa‐miR1866‐3p in serum. qRT‐PCR was performed to determine levels of osa‐mir1866‐3p in serum across all diets at Days 0 and 28. No treatment, time, or time by treatment effect was found. *p*‐value ≤ .05 shown as group means and SD. Data were normalized to RNU1.

**FIGURE 4:**
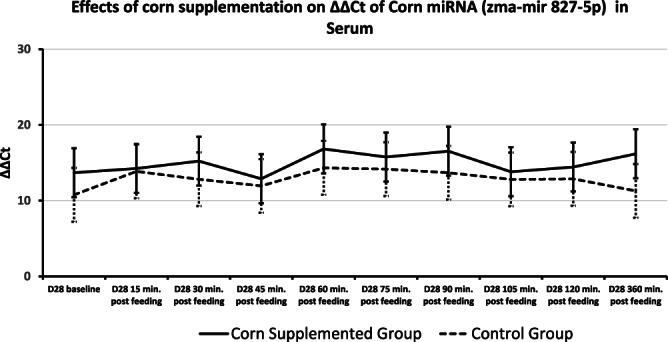
Level of corn miRNA (zma‐miR827‐5p) in serum across time. qRT‐PCR was performed to determine levels of zma‐mir 827‐5p in serum across all diets at Days 0 and 28. A treatment effect for the corn group was found (*p* < .05). No time or time by treatment effect was found. *p*‐value ≤ .05. Shown as group means and SD. Data were normalized to RNU1.

Plant miRNA transcripts (ath‐miR156a, zma‐miR827‐5p, and osa‐miR1866‐3p) were detectable in total serum miRNA on Days 0 and 28. There was a time effect for ath‐miR156a, where ΔΔCt increased from 15 min after sampling to 45 min after sampling returning to 15‐min levels at 120 min after sampling (Table 4, Figure 2). Rice miRNA, osa‐miR1866‐3p, was detected in all horses at each time point and no treatment, time, or time by treatment effect was found (Figures 2 and 3). There was a treatment effect for corn miRNA. Zma‐miR827‐5p was detected in horses from both groups with horses fed corn having overall greater changes in serum levels of Zma‐miR827‐5p ΔΔCt (Corn‐supplemented group = 14.96 ± 0.64; control group = 12.85 ± 0.64) of this miRNA transcript (*p* < .054), though no time or time by treatment effect were found (Figure 4). All three plant miRNA transcripts (ath‐miR156a, zma‐miR827‐5p, and osa‐miR1866‐3p) were detected in the muscle samples of each group, and no time or treatment effects were found (data not shown).

**TABLE 4:** Effects of corn supplementation on ΔΔCt of plant miRNA (ath‐mir 156a) in serum.

ath‐mir 156a	Pre	15 min	30 min	45 min	60 min	75 min	90 min	105 min	120 min	360 min
Combined trt	7.02 ± 3.95^a^	9.15 ± 3.78^ac^	8.22 ± 3.95^ac^	10.78 ± 3.78^bc^	10.95 ± 3.78^bc^	10.71 ± 3.78^bc^	11.24 ± 3.78^bc^	11.13 ± 3.78^bc^	6.86 ± 3.78^a^	7.86 ± 3.78^a^

*Note*: qRT‐PCR was performed to determine levels of ath‐miR156a in serum across all diets at Days 0 and 28. ΔΔCt is represented as the mean difference between baseline and Day 28, plus or minus SE. Time effect *p* = .0062. No treatment or time by treatment effect was found. *p*‐value ≤ .05. Data were normalized to RNU1. Different letter superscript mean different values over time (*p* < .050).

### 3.3 Equine endogenous miRNAs

A total of 41 equine miRNA transcripts were detected at all time points in all serum samples. Analysis of the equine serum samples found a significant treatment effect (*p* < .05) for 12 of the 41. Seven of the miRNA transcripts related to metabolic disorders (Table 5), while the other five transcripts related to other diseases. Eleven of these 12 miRNA transcripts were significantly upregulated in the CORN group compared with the CONTROL group, while eca‐mir192 showed a downregulation in CORN group compared with CONTROL (Tables 5 and 6). Analysis of eca‐mir129a5p revealed a time‐by‐treatment effect, in CONTROL samples 1‐h post‐feeding where CORN increased from baseline at 60 min and decreased from 60 to 360 min and CONTROL decreased from baseline remaining stable from 60 to 360 min, where it had lower levels compared with the CORN samples (Table 5). A time effect was also found on day 28 1‐h after feeding for eca‐mir4865p, and both groups had downregulation of this miRNA transcript at this time point compared with Day 0, from 60 to 360 min (Table 5).

**TABLE 5:** Significant difference in endogenous miRNAs related to obesity or metabolic disorders within serum from Days 0 to 28.

Difference between day 0 and 28	Treatments *n* = 6 per group			*p*‐value		
ΔΔCt	Corn	Control	SEM	TRT	TIME	TRT*TIME
ecamir‐16 trt	6.30^a^	0.89^b^	1.57	.036	.27	.76
60 min	6.66	1.52	1.69	–	–	–
360 min	5.94	0.26	1.69	–	–	–
ecamir‐4893p trt	3.35	−0.13	0.99	.032	.17	.44
60 min	3.69	1.01	1.21	–	–	–
360 min	1.03^a^	0.55^b^	1.21	–	–	–
ecamir‐4865p trt	7.00^a^	1.72^b^	1.52	.034	.040	.66
60 min	8.31	3.82	1.83	–	–	–
360 min	5.68	−0.39	1.83	–	–	–
ecamir‐126‐3p trt	1.41^A^	−0.33^B^	0.49	.031	.52	.66
60 min	1.46	−0.041	0.62	–	–	–
360 min	1.36	−0.62	0.62	–	–	–
ecamir‐296 trt	3.86^A^	−0.59^B^	1.11	.017	.081	.98
60 min	4.80	0.31	1.29	–	–	–
360 min	2.93	−1.49	1.29	–	–	–
ecamir‐192 trt	0.29^A^	6.15^B^	1.42	.016	.068	.23
60 min	6.56	32.07	1.61			
360 min	5.73	−1.49	1.61			
ecamir‐129a5p trt	1.03	0.09	0.54	.26	.66	.039
60 min	1.57	0.47	0.61			
360 min	−0.28	0.46	0.61			

*Note*: qRT‐PCR was performed to determine levels of endogenous miRNAs in serum across both diets at Days 0 and 28 1 h and 6 h post‐feeding. Represented as the mean difference between baseline and Day 28 1‐h post‐feeding and 6 h post‐feeding. Presented as means and standard error of the means. *p*‐value ≤ .05. Values within a column lacking a common superscript differ, by *p* ≤ .05. Control: forage only supplemented horses; Corn: corn‐supplemented horses.

**TABLE 6:** Significant difference in endogenous miRNAs within serum from Days 0 to 28.

Difference between day 0 and 28	Treatments *n* = 6 per group			*p*‐value		
ΔΔCt	Corn	Control	SEM	TRT	TIME	TRT*TIME
ecamir‐195 trt	8.00^A^	0.63^B^	2.01	.027	.10	.38
60 min	8.51	2.24	2.18	–	–	–
360 min	2.93	−0.98	1.29	–	–	–
ecamir‐326 trt	5.40^A^	0.039^B^	1.49	.030	.54	.72
60 min	6.02	0.21	1.74	–	–	–
360 min	4.78	−0.13	1.74	–	–	–
ecamir‐3715p trt	2.89^A^	−1.41^B^	1.18	.039	.44	.74
60 min	3.58	−1.14	1.54	–	–	–
360 min	2.21	−1.69	1.54	–	–	–
ecamir‐1271 trt	2.74^A^	−1.61^B^	0.49	.0084	.35	.44
60 min	2.83	−2.51	1.18	–	–	–
360 min	2.66	−0.62	0.62	–	–	–
ecamir‐4903p trt	7.99^A^	0.63^B^	2.01	.027	.10	.38
60 min	8.51	2.24	2.18			
360 min	5.73	−0.98	2.18			

*Note*: qRT‐PCR was performed to determine levels of endogenous miRNAs in serum across both diets at Days 0 and 28 1 h and 6 h post‐feeding. Represented as the mean difference between baseline and Day 28 1‐h post‐feeding and 6 h post‐feeding and SE. No time or time by treatment effect was found. *p*‐value ≤ .05. Values within a column lacking a common superscript differ, by *p* ≤ 0.05. Trt treatment effect, time time effect, trt*time treatment by time effect. Control: forage only supplemented horses; Corn: corn‐supplemented horses.

Sixteen miRNA transcripts were detected in the muscle samples of most horses at both time points. The analysis found a treatment effect for eca‐mir106b after corn supplementation (*p* = .057). CORN ΔΔCt was 12.97 ± 3.92 and CONTROL ΔΔCt was 1.04 ± 3.51. No time or time‐by‐treatment effects were found.

## 4 DISCUSSION

This study aimed to detect diet‐derived plant miRNA transcripts within equine serum and skeletal muscle and to examine the impact of corn supplementation on profiles of endogenous miRNAs in circulation and skeletal muscle. To the authors' knowledge, this study is one of the first to demonstrate dietary impacts on endogenous equine miRNA expression in serum. The results presented here highlight the complex interactions that exist between diet and miRNAs.

### 4.1 Diet‐derived plant miRNAs

The CT of corn miRNA (Zma‐miR827‐5p) is relatively high indicating low concentrations of this miRNA in corn, similar to a previous study (Luo et al., 2017). The presence of plant miRNA transcripts (ath‐miR156a, zma‐miR827‐5p, and osa‐miR866‐3p) in total serum samples is consistent with results from previous studies in mice, humans, and cattle (Wang et al., 2012; Zhang et al., 2012), while contradictory to other studies in mice (Dickinson et al., 2013) and horses (Nulton, 2014) that did not find plant miRNAs in total serum 360 min after feeding. Previous research examining the uptake of dietary miRNAs into circulation shows that many are inconsistently absorbed or detected with variation described between individual animals (Yang et al., 2015). The detectability of plant miRNAs in circulation varies greatly which could be due to re‐uptake of the miRNAs into tissues, individual variation in the absorption of intestinal contents, and extremely low abundances of transcripts. However, it is exciting that even at those levels changes in endogenous miRNA can be found. Further research needs to be conducted to better understand if and how miRNAs present in the diet make it into the bloodstream. Our current study detected both ath‐miR156a and osa‐miR18663p in total serum from all tested horses with no difference between the dietary treatments, or time effect for osa‐miR18663p and no time‐by‐treatment effect for both miRNAs. However, a time effect for ath‐miR156a showed an increase from baseline to 45 min post feeding the CORN and fasting in the CONTROL group and return to baseline levels at 120 min. Despite fasting for the CONTROL group and corn ingestion in the CORN group, the increase in alt‐miR156 occurred in both groups. Horses had free access to hay during the hours preceding the blood collection. This appearance in plant miRNA could reflect plant material being absorbed and increasing in the blood between 45 and 120 min and then returning to baseline levels. The corn miRNA (zma‐miR827‐5p) was present in both treatment groups before corn supplementation with much greater levels detected in the serum samples from the CORN treatment group after supplementation. In equine digestibility studies looking at steam‐flaked corn glucose levels began rising post‐ingestion by 30 min and peaking at an average of 94 min post ingestion (Vervuert et al., 2004). Since ΔΔCt of zma‐miR827‐5p showed an upregulation compared with before supplementation period, the treatment effect found for corn fed horses suggests that miRNAs can be absorbed into the circulation within the early segments of the small intestine.

The reason for detecting the corn and rice miRNAs before supplementation is not clear, however, the differences from Days 0 to 28, at different times, as analyzed by ΔΔCt show an increase from before supplementation started. All study horses were on a forage‐only diet in the 21 days before Day 0 samples and to our knowledge were not supplemented with anything during this adjustment period. The horses were, however, located on the same premises and had intermittent access to the same pens as horses receiving grain containing corn and rice bran for an unrelated study (Catandi et al., 2020), suggesting a possible source of the corn and rice residues. Quantitative RT‐PCR is highly sensitive and specific for detecting miRNAs which may have allowed for the corn and rice residues to be detected in our study (Balcells et al., 2011). Since assays are very sensitive, residues can be detected in serum and muscle.

All plant miRNAs (plant, corn, and rice) tested were detectable in equine muscle samples at Days 0 and 28, but no time, treatment, or time‐by‐treatment effects existed. The presence of plant miRNAs in tissue is in line with other studies that have found plant miRNAs to be taken up into mammalian tissues (Luo et al., 2017; Zhang et al., 2012). In contrast to Nulton (Nulton, 2014) which found only ath‐miR156a present in equine tissue samples regardless of diet, our study identified zma‐miR827‐5p and osa‐miR18663p in equine muscle tissue in addition to ath‐miR156a. As previously mentioned, another study conducted at the same time as our study could explain the presence of osa‐miR18663p in our samples despite our diets not containing rice. The concurrent study could also explain the presence of these plant‐derived miRNAs in our samples before the start of the feeding trial. As the impact of feeding corn on miRNA presence in horses was the main focus of this study, we had a special interest in the potential presence of corn miRNA in skeletal muscle. It is possible that a larger amount of corn should be fed to horses in future studies to produce a detectable effect and specific care should be taken to avoid environmental cross‐contamination. A longer supplementation time study could also clarify the results.

Our study shows that plant‐derived miRNA transcripts can be detected in equine serum and tissue after ingestion, but more equine studies should be conducted to confirm these findings as most studies investigating this phenomenon have been conducted in mice, pigs, and humans which are physiologically different than horses, especially regarding the digestive tract, making comparisons between studies hard. The specific plant miRNAs used in this study are not heavily used by other research studies in this area, so direct comparison is not possible for many studies, however, the ability to absorb diet‐derived miRNA likely is not impacted by the individual miRNA itself. The specific plant miRNAs in our study were chosen because they were used in the previous equine study (Nulton, 2014). The primers for corn, plant, and rice miRNA are highly specific and no other primers could yield similar results. Continued research into the mechanism by which diet‐derived miRNAs exit the intestinal lumen and the form in which they are present in circulation, either as free miRNAs or encapsulated in micro‐vesicles such as exosomes is needed. The ability of plant miRNAs to be absorbed into circulation and tissues and determining the levels required per cell to have an impact on mammalian gene regulation holds the potential for new therapeutic or diagnostic techniques to be developed within horses and other mammals.

### 4.2 Impact of diet on equine endogenous miRNAs

The results showed 12 miRNAs in serum were impacted by corn supplementation of which six, eca‐mir16, ‐4863p, ‐4865p, ‐126‐3p, ‐296, and ‐192, have orthologs linked to obesity or metabolic disease. Meerson et al. (2019) found mir16‐2‐3 and mir126‐5p could be used to distinguish early from complicated T2DM in human individuals with 77% accuracy, these two miRNAs are in the same family as mir16 and mir126‐3p, respectively, suggesting that these families could serve as biomarkers for metabolic diseases similar to T2DM. The human miRNA mir126‐3p has also been found to have decreased levels in plasma and circulating angiogenic cells of patients with T2DM compared to healthy individuals, however, amongst subjects with T2DM those with a history of a major cardiac event had lower levels of mir126‐3p, which suggests it can serve as a biomarker for systemic inflammation and angiogenic status (Olivieri et al., 2015). Our study found the equine homolog of mir126‐3p to be upregulated CORN serum samples compared with the CONTROL samples. Another study showed that patients with T2DM that had good glycemic control had higher mi126‐3p compared to poor glycemic control in T2DM patients (Olivieri et al., 2015). The higher levels in mir‐126‐3p on CORN fed horses could just indicate the body's response to the higher glucose levels that occurs after starch feeding. We did not measure blood glucose, but corn ingestion leads to increases in glucose in horses (Vervuert et al., 2004). Our study found eca‐mir4863p upregulated in the CORN samples compared with the CONTROL samples, similar to Nulton's study which found this transcript to be increased in horses fed corn (Nulton, 2014). In addition, previous studies have found circulating mir4863p in humans to be increased in T2DM patients and obese children (Marzano et al., 2018; Meerson et al., 2019; Prats‐Puig et al., 2013). Horses fed CORN showed higher levels of eca‐mir4865p at 1‐h post‐feeding compared to CONTROL. This miRNA also decreased with time between 60 and 360 min in both groups. Eca‐mir296 was upregulated in the CORN serum samples compared with CONTROL. Studies of human ortholog, mir296, demonstrated higher concentrations of this transcript in the visceral adipose tissue of obese individuals compared with non‐obese, with an apparent relative decrease in expression levels in the obese subjects with T2DM (Gentile et al., 2019). Although this study (Gentile et al., 2019) linked mir296 to the regulation of adipose tissue through the WNT pathway it did not directly investigate circulating miRNAs. Another miRNA found within our study with an ortholog linked to obesity and related disorders is eca‐mir192, which we found downregulated in the CORN. Previous studies have proposed mir192 as a conserved biomarker for obesity as it was found to have increased levels in the circulation of both mice and humans suffering from obesity (Jones et al., 2017; Ortega et al., 2013). Multiple studies have also found increased abundance of mir‐192 in the exosomes from individuals with insulin resistance, suggesting this transcript could play a role in the disease (Jones et al., 2017; Párrizas et al., 2015; Shah et al., 2017). Mir192‐5p which is in the same family as mir192 has been found to regulate lipid synthesis in non‐alcoholic fatty liver disease, which can develop as the result of metabolic disorders, and to be downregulated in patients with diabetic‐related kidney disease (Assmann et al., 2018; Liu et al., 2010). The studies associating mir192 with obesity, metabolic disorders, insulin resistance, and a possible role in lipid synthesis suggest that mir192 should be included in future studies looking at these disorders across species.

In our study, eca‐mir129a5p was upregulated in the CORN samples. In a previous study, eca‐mir129a5p was found to have greater levels in circulation within insulin‐resistant horses compared to insulin‐sensitive horses (da Costa Santos et al., 2018). We did not test the horses for insulin resistance so we cannot say for sure that the upregulation of ecamir‐129a5p found at 1‐h post‐corn supplementation suggests these horses may have been at a higher risk of developing insulin resistance as a result of corn supplementation. Future studies measuring parameters related to insulin resistance prior to and after corn supplementation in addition to eca‐mir129a5p levels could shed light on if a connection exists. It could be more informative if the future study determines glucose tolerance test (IVGTT), as genetic analysis alone is not sufficient for accurate findings because gene expression is very sensitive and needs to be confirmed by other physiological parameters.

Ecamir‐195 was higher in CORN compared with CONTROL horses. The human ortholog miRNA has been associated with cancer control when in an upregulated state (Liu et al., 2010). Mir 326 has been associated with tumor growth suppression (Ghaemi et al., 2019). Mir 1271 has also been associated with tumor growth suppression (Xiang et al., 2015). We could not find literature on the two remaining miRNAs in the table (ecamir‐3715p, and ecamir‐4903p).

Analysis of the muscle samples found eca‐mir106b to have a significant treatment effect, which has orthologs linked to obesity or muscle insulin response. Within insulin‐resistant skeletal muscle, mir106b was found to be upregulated and contribute to the regulation of skeletal muscle glucose uptake (Zheng et al., 2019). Regulation of glucose uptake by this miRNA is due to its regulation of the genes coding for mitofusin‐2 (Mfn2) and glucose transporter (GLUT)‐4 resulting in reduced levels of these proteins, reduced insulin sensitivity, and increased blood glucose concentrations (Zhang et al., 2013, 2017).

Our study illustrates that diet does have a role in regulating levels of endogenous miRNAs in circulation and skeletal muscle of horses. The daily corn ration for each horse, 453 g, was a relatively small amount compared with the amount of concentrate given to some horses and likely a larger amount of corn may be needed to see larger impacts. It should also be noted that making comparisons between studies can be difficult as the physiology of horses differs from humans and mice, this could also contribute to differences in findings. Also, there are other corn specific miRNAs that could be used for the detection of corn absorption as shown before (Luo et al., 2017). More equine studies should be conducted within this area using larger sample sizes, higher corn content diets, longer feeding times, and potentially including animals with known insulin resistance or predisposing factors to better understand how much of an impact high NSC diets can have on miRNA expression.

## 5 CONCLUSIONS

Based upon the results from the current study we conclude that plant miRNAs can be absorbed, released into circulation, and taken up by muscle tissue in horses. These findings support the possibility of interspecies gene regulation (Zhang et al., 2012). The ability of diet‐derived miRNAs to be absorbed and identified within total serum and muscle tissue is a major step in the process of gaining a better understanding of the mechanisms involved in the uptake of diet‐derived miRNAs and the roles they may play in gene regulation.

## FUNDING INFORMATION

This research was partially funded by the Y‐Cross Ranch Animal Agriculture Scholarship Fund through their Y‐Cross Ranch Davis Scholar program supporting C. Carver.

## CONFLICT OF INTEREST STATEMENT

The authors declare that they have no conflict of interest.

## ETHICAL APPROVAL

All experiments were approved by the Colorado State University Institutional Animal Care and Use Committee (Protocol #18‐7961A). The experiments were performed in compliance with the US National Research Council's “Guide for the Care and Use of Laboratory Animals”, the US Public Health Service's “Policy on Humane Care and Use of Laboratory Animals”, and “Guide for the Care and Use of Laboratory Animals”.

## Data Availability

The data that support the findings of this study are available from the corresponding author upon reasonable request.
